# Ethical issues in denial of church wedding based on couple’s hemoglobin genotype in Enugu, south eastern Nigeria

**DOI:** 10.1186/s12910-019-0376-8

**Published:** 2019-05-29

**Authors:** Euzebus C. Ezugwu, Pauline E. Osamor, David Wendler

**Affiliations:** 1 Department of Obstetrics & Gynecology, Faculty of Medical Sciences, College of Medicine, University of Nigeria, Ituku-Ozalla Campus, PMB 01129, Enugu State, 400001 Nigeria; 2 Department of Bioethics, Clinical Center, National Institutes of Health, Bethesda, MD 20892-1156 USA

**Keywords:** Hemoglobin genotype, Church wedding, Ethics, Denial, Nigeria, Sickle cell anemia, Premarital screening

## Abstract

**Background:**

Sickle cell anemia (SCA) is a major genetic disease with the greatest burden in sub-Saharan Africa. To try to help reduce this burden, some churches in Nigeria conduct premarital sickle cell hemoglobin screening and refuse to conduct weddings when both individuals are identified as carriers of sickle cell trait.

**Main body:**

This paper explores the ethical challenges involved in such denials. We assess whether churches have the right to decline to marry adults who understand the risks and still prefer to get married, and whether couples should be denied church weddings based on the risk that their child may suffer from sickle cell anemia. We examine the moral and ethical dimensions of such denials and explore the underlying socio-cultural context involving the purpose of marriage and the meaning of the wedding ceremony in societies where premarital screening is one of the few tools available to reduce the risk of having children with SCA. The potential role of the church is also examined against the background of church beliefs, the duty of the church to its members and its role in reducing the suffering of its members and /or their children.

**Conclusion:**

We argue that the church should impose these burdens on couples only if doing so promotes a sufficiently compelling goal and there is no less burdensome way to achieve it. We then argue that the goal of reducing the number of individuals in Nigeria who have SCA is compelling. However, testing earlier in life offers a less burdensome and potentially even more effective means of achieving this goal. This suggests that, advocating for earlier screening and helping to support these programs, would likely better promote the church’s own goals of helping its parishioners, increasing the number of church weddings, and reducing the burden of SCA in Nigeria.

## Background

Sickle cell anemia (SCA) is an autosomal recessive blood disorder [1]. Carriers who have one copy of the mutant hemoglobin S gene typically have no symptoms. When both parents are carriers, there is a 25% chance that a pregnancy will result in a child who is affected with sickle cell anemia (Hemoglobin SS). Affected individuals develop red blood cells that become sickled in shape, resulting in cells which block small blood vessels, reducing the flow of blood through the vessels and damaging the tissue that is served by them. The resulting pain can be terribly bad and can interfere with many aspects of the patient’s life, including education, employment and psychosocial development [2]. In severe cases, massive destruction of red blood cells can lead to severe anemia and even death.

Sickle cell anemia is seen in the Arab world [3] and in Western countries, where it affects predominantly people of black African and Caribbean origin [2]. However, the vast majority of SCA occurs in sub-Saharan Africa, which accounts for approximately 75% of the 300,000 global SCA births every year, with an estimated 50–80% of affected children dying before adulthood [4]. In Nigeria, it is estimated that about 150,000 children are born annually with SCA, with a prevalence rate of 20 per 1000 livebirths [5]. Approximately 24% of Nigeria adults are sickle cell carriers [5]. In Enugu, south east Nigeria, and Kano, north west Nigeria, prevalence rates of 22 and 40.5% respectively have been reported [6, 7].

SCA is one of the leading inherited risk factors for childhood death in Nigeria and affected children historically have been referred to as “malevolent Ogbanje” because it was believed they will be reincarnated and die again to cause further sorrow for the family [8]. With advances in knowledge about the cause and clinical characteristics of SCA, myths and misconceptions surrounding the disease are being slowly dispelled. Interestingly, unlike some inheritable genetic disorders, sickle cell carriers are considered normal and do not suffer any form of discrimination or stigmatization in Nigeria.

Bone marrow transplantation offers a potential cure for SCA [9, 10]. However, it is very expensive and largely unavailable in Nigeria. Indeed, the first ever bone marrow transplant in Nigeria was only recently reported, at University of Benin Teaching Hospital [11, 12]. Efforts in Nigeria have thus focused on prevention. Pre-natal diagnosis and the termination of affected pregnancies is possible, but is widely regarded as ethically and religiously problematic in Nigeria [13]. Abortion is also illegal in Nigeria unless the life of the mother is at risk [14]. As a result, premarital screening, that is, screening engaged couples prior to the wedding, has become a key strategy for the prevention of SCA in Nigeria.

Premarital screening for SCA has been adopted by many churches in Nigeria. A study conducted in 2004 found that premarital sickle cell trait screening was a pre-condition for a church wedding in 58.8% of cases [14]. The percentage may be even higher today as many churches in Nigeria (especially in the south eastern part of Nigeria) include premarital screening for SCA as part of the premarital counselling process and often insist on reviewing the test results before conducting the wedding. When both individuals are sickle cell carriers, the church discourages them from marrying. Some church denominations, especially in Enugu state, go further and refuse to wed couples when both individuals are sickle cell carriers.

In many Nigerian cultures, marriage is officially contracted by the payment of the bride price by the bridegroom to the bride’s family. In Iboland, in south eastern Nigeria, once this is done, the couple are regarded as married. However, couples from Christian families are expected to wed in the church. Those who fail to do so are seen as committing a sin by the church and experience strong social disapproval. As a result, Christian couples in Nigeria have a strong interest in a church wedding and the efforts of churches to discourage sickle cell carriers from marrying, and even denying them a church wedding, represents a significant dis-incentive to marriage.

While it is unclear exactly what percentage of couples get married against church advice, many experience pressure in this regard and some likely decide to abandon the relationship. The church’s approach thus has the potential to decrease the prevalence of SCA and thereby decrease the burden of SCA on families and the health care system. This approach also can pose significant costs on the couples affected. It can lead to those who choose not to wed having to live without their chosen partner and couples who choose to wed outside of the church living with stigmatization. The goal of the present paper is to explore this ethical tension that arises when churches discourage couples from marrying and even deny them a church wedding.

## Main text

### Premarital screening as a strategy for prevention of inherited disorders

Premarital screening involves couples who plan to wed undergoing a panel of tests for “genetic, infectious and blood transmitted diseases to prevent any risk of transmitting any disease to their children” [15]. Premarital screening appears to have started in Virginia in 1970, with testing for SCA [16]. This was followed 5 years later by the first recorded premarital screening for thalassaemia, another inherited blood disorder, in Italy [17]. Premarital screening for thalassemia is now common in Cyprus, Greece and Italy [18]. It is also used for the prevention of other genetic disorders, including congenital anomalies and hemoglobinopathies (for which premarital screening is mandatory in Saudi Arabia) [3].

Criteria for screening in general were first proposed by Wilson and Junger in 1968 [19]. These criteria were adopted by World Health Organization (WHO) with some modifications to take into account the rapidly growing field of genetics. The WHO guidelines support screening under the following conditions:

(i) The screening programme should respond to a recognized need. (ii) The objectives of screening should be defined at the outset. iii) There should be a defined target population, (iv) There should be scientific evidence of the effectiveness of screening. (v) The programme should integrate education, testing, clinical services and programme management. (vi) There should be quality assurance, with mechanisms to minimize potential risks of screening. (vii) The programme should ensure informed choice, confidentiality and respect for autonomy. (viii) The programme should promote equity and access to screening for the entire target population. (ix) Programme evaluation should be planned from the outset. (x) The overall benefits of screening should outweigh the harm [20].

Based on these criteria, SCA in Nigeria appears to qualify for screening. SCA is clearly a disease of public health importance in Nigeria, which has facilities for diagnosis. There is no treatment available, other than attempts to manage the symptoms, and the disease cannot be prevented in affected individuals. Premarital screening and discouraging carriers from marrying provides a way to reduce the number of affected children and thereby reduce the associated public health burden.

Premarital screening is aimed at identifying intending couples where both individuals are carriers and counselling them. Premarital screening involves educating the couple and offering them accurate and unbiased information to assist them in making an informed decision. It is hoped that, with proper counselling and education, most intending couples will discontinue the relationship, avoid having children, or adopt children. The level of awareness and acceptance of pre-marital genetic screening for SCA in Nigeria appears to be high. There are reports of an awareness rate of 50.7% among youth in Tina community of Plateau state [21], 90.5% among national youth service corps member in Owo Ondo state [22], and 84.4% among married couples in a community in Rivers state [23].

Oyedele et al. reported a premarital screening acceptance rate of 77.3% in Plateau state [21] and Gbeneol et al. found that married couples in Choba, Rivers State have a positive attitude towards premarital screening [23]. In that study, 72.8% of the respondents had premarital screening before marriage, with 88.97% recommending that pre-marital screening be made compulsory.

### Should the church refuse to conduct weddings for individuals at high risk of having children with SCA?

The concept of a flourishing life refers to a life that is good for the individual whose life it is, and has been described as follows:

To flourish is to lead the sort of life it is good to lead, by which is meant the sort of life you want your children to lead, as well as the sort of life you want to lead yourself. Such a life is not just good in the sense that it is good that someone should lead such a life—it may be good that someone leads a life of self-sacrifice without that person’s life being the sort of life it is good to lead in the relevant sense, good for him or her [24]

Since the time of the Ancient Greeks there has been significant debate over the constituents of a flourishing life. Is it better for individuals to contemplate philosophical truths or spend time with their friends? Better to have children and contribute to the next generation, or spend one’s money on seeing the world. Marriage is widely regarded as central to a flourishing life to the extent that individuals have a fundamental interest in being able to marry. The impact of marriage on the extent to which one has a flourishing life depends critically on the choice of one’s partner. In addition, individuals tend to be better judges of their own interests and have a strong motivation to choose a partner suitable for them. This suggests, absent a wise and benevolent matchmaker, that individuals have a strong interest in choosing for themselves whom they marry. Arguably, these interests are sufficiently strong that individuals have a right to marry, including a right to choose whom to marry, in the sense that others should not interfere with competent adults marrying unless doing so promotes a compelling goal and there is no less intrusive way to attain that goal.

One might try to support the church’s current approach by denying that marriage is central to a flourishing life. If one accepted this view, there would be little need to justify the church’s process of premarital screening. However, the church itself does not endorse this approach. Instead, the church encourages the view that marriage is central to a flourishing life and adults with the capacity to make an informed decision should be able to decide whom to marry. This suggests that the church accepts the view that individuals have a strong interest or a right to marriage. Hence, even on the church’s own terms, we can ask whether its current approach to premarital screening is consistent with recognition of this fundamental interest or right.

If the *state* prohibited couples from marrying, it arguably would violate their right to marry. In contrast, while it seems plausible to argue that competent adults have a right to choose whom to marry, they do not have a right to be married wherever they prefer. Certainly, a couple cannot insist on being married in a specific church, especially when the church disagrees with the marriage for understandable reasons. Prohibition on marriage in the churches of an entire region places more burden on the couple, although it does not prevent them from marrying outside the church (such as at a registry).

At the same time, the church actively promotes the view that a church wedding legitimizes a marriage. Hence, its own efforts foster the environment in which marriage outside the church is stigmatized, thus placing significantly greater costs on those who choose this option. This suggests that, while the church’s approach may not, strictly speaking, violate the rights of the couple, it places significant burdens on them, and should be pursued only with strong justification and in the absence of a less burdensome alternative.

Two persons who know that they are SCA carriers and choose to marry are putting their future offspring at significant risk of SCA. Individuals have a right to make this decision. However, avoiding this risk avoids having children who suffer the pain of SCA, frequent illness and hospitalizations and who may experience an early death. It is also very difficult for parents to raise children with SCA, especially in resource poor settings where effective symptomatic care may not be readily available. The economic burden can be significant, creating a ripple effect on the entire family and, in aggregate, on society. Finally, a lower incidence of SCA in the country would place fewer burdens on a health care system that is already unable to provide sufficient care for all who need it. These considerations provide strong support for the program of discouraging and even refusing to conduct weddings for couples who are SCA carriers.

Since the church is favorably disposed to child adoption, one might argue that the church could instead agree to marry individuals who are both carriers on the condition that the couple agrees not to have any biological children and to adopt if it wants to have children at all [25]. While this approach seems reasonable, the church may be reluctant to pursue it on the grounds that it lacks the capacity to ensure that the couple abides by their agreement. In addition, having biological children is central to most marriages in Nigeria. In traditional African homes, a woman is not considered the mother of the house until she bears a child (and in many cases, a son). And a woman who does not bear a child within a few years of marriage stands the risk of being divorced [26–29]. Any proposals for alternative approaches to the church’s screening process should take these aspects of the culture into account.

### Timing of screening

Currently, screening and counseling of sickle cell carriers occurs prior to the wedding. In other words, it often occurs after the two individuals have been dating, possibly for years, and are engaged to be married. By this time in Nigeria, the two families would also have met and given their approval to the proposed union. Therefore, raising the obstacle of no church wedding at this stage can place significant stress on the couple. This approach also may be less effective in achieving the goal of reducing SCA. Couples who have been dating for a prolonged period and are attached to each other to the point of intending marriage are likely to be disinclined to end the relationship. Fortunately, it appears that these burdens may be avoidable. In particular, it is in principle possible to screen individuals earlier in life and thereby reduce the chances that two sickle cell carriers will end up committed to one another in the first place [4, 17, 19].

Earlier screening would provide SCA carriers with the information necessary to make informed decisions when entering in relationships rather than providing this information after the relationship has led to a commitment to marriage. The central question here is when the screening should be done. Many have argued that genetic testing should be delayed when possible until the individuals are adults and can decide for themselves whether to be tested and learn their status. This approach has the virtue of allowing individuals to decide for themselves whether they undergo genetic testing and, if so, for what are they tested. It is especially suited to conditions which do not manifest until adulthood, such as Alzheimer disease, which typically does not manifest until the 6th decade of life or later.

The problem with delaying testing in the present case is that in Nigeria many people commit to one another in the teenage years. According to the 2013 Nigeria National Demographic Health survey (NDHS), half of women ages 25–49 years were married by the age of 18 years and 61% by the age of 20 years [30]. This suggests that the process of committing to a specific partner begins in the early to mid-teens for many individuals in Nigeria. While attempts to discourage or forbid a church wedding among carriers may be effective in some cases, it is likely that other couples will still marry given their strong commitment to each other. A study in Saudi Arabia found that over 80% of intending couples married despite knowing that they were at high risk of having children with SCA [3]. A small study conducted by Gbeneol et al. found that of 19 couples who were both sickle cell carriers, 4 (21.05%) chose to marry, believing in the power of God to keep them from having a child with sickle cell disease (*n* = 3) or citing difficulty ending the relationship (*n* = 1, 23]. While it is unclear how generalizable these data are, they suggest that waiting until prior to the wedding may not dissuade some couples who are carriers from marrying in Nigeria. In addition, some percentage of these are likely to choose to marry outside the church. In these cases, the present approach undermines the church’s goal of encouraging church wedding.

One option for avoiding these concerns would be to screen individuals in early adolescence. The advantage of this approach is that the individuals would be old enough to have some understanding of the process. An alternative approach would be to incorporate screening for sickle cell disease and carrier status into infant welfare and under-five child health programmes. One advantage of this approach is that these programs are already in place in several programmes and locations in Nigeria. For example, the Institute of Child Health in Ibadan, south west Nigeria has been doing infant screening for sickle cell anemia and carriers for decades [31]. Such programmes would need to be expanded to become true population screening programmes. Although neonatal screening for Sickle cell disease (SCD) and carriers is recommended in the recently launched revised Nigeria national health policy document [32], the government is yet to establish a national sickle cell neonatal screening programme to effect this policy. The Government might establish designated places where hemoglobinopathy screening, including carrier status, can be done free of charge to all citizens and a medical certificate indicating their hemoglobin type issued to every screened child. Also, screening for SCA could be included as part of the investigation done under medical examination for fitness during entrance into primary and secondary schools and other opportune times during the adolescent period.

### Implementation and assessment of the present proposal

On the proposed approach, the role of the church would change, it would not be eliminated. Instead of requiring screening and providing counseling at the time a marriage is being considered, the church could focus on encouraging parents to screen their children and inform the children about their hemoglobin status early in life. This approach would drastically reduce the number of couples who find out about their risk of having a child with SCA only after they have committed to wed. In addition, the church might focus on teaching individuals early in life about the personal and societal costs of SCA and encouraging carriers to seek out partners who are not themselves carriers. In this regard, the fact that sickle cell carriers are not stigmatized is important. It decreases concern that identifying carriers earlier in life may cause problems for them.

We believe that the present proposal offers the potential to better promote the church’s own goals. This suggests that the present proposal is not foreign to nor in tension with the church’s principles. In particular, the church accepts that marriage is a fundamental part of a flourishing life. The church also believes that marriage in the church is important. At the same time, the church presumably wants to minimize the burdens it places on its parishioners and has the explicit goal of reducing the number of children born with SCA. The present analysis suggests that the proposed approach is likely to promote these goals more effectively than the present approach of screening couples just prior to the wedding. In our view, another significant advantage of screening early in life is that the results could be incorporated into the individual’s understanding of themselves and their life trajectory, rather than having to adjust to this information later on. In particular, individuals who are informed from an early age that they are carriers could make decisions regarding their relationships with this information in mind. Future studies exploring the psychosocial outcomes of these children would be beneficial in informing this discussion.

The proposed approach seems consistent with the Dor Yeshorim program for screening for Tay Sach diseases, a similar hereditary genetic disease. The Dor Yeshorim program is an anonymous, highly confidential early premarital screening for Tay Sach disease, targeted towards young people (high school girls and seminary boys) in orthodox Jewish communities in an effort to prevent the disease and at the same time avoid the stigma associated with an individual’s carrier status becoming known. This early screening program helps intending couple know whether they are compatible or not before committing to marriage, but individuals’ carrier status is not disclosed. However, the choice of whether or not to pursue marriage based on this information is strictly up to the couple [33]. Interesting, unlike Tay Sach disease, sickle cell carriers are not stigmatized in Nigeria.

While the proposed approach makes sense in theory, future empirical and sociological research would be valuable to assess its impact in practice. This research could provide valuable insight into how to deal with inherited diseases more generally in low resource settings. In particular, it will be important to assess the impact of being identified as a sickle cell carrier early in life. What is the psychological impact of this information? Does this information increase the extent to which sickle cell carriers seek out partners who are not sickle cell carriers. How can the church most effectively be a part of this process?

Finally, while this alternative approach would allow the church to promote the goal of fewer individuals in Nigeria with SCA, while reducing the burden on individuals who are carriers, this approach depends on the state instituting a program of early child testing for SCA status. The church could be strong advocates for developing such programs, both as a way to benefit all Nigerians and as a means of reducing the burdens it places on its parishioners while pursing laudable goals.

## Conclusions

Premarital screening for SCA carriers is increasingly common in south east Nigeria and some churches deny weddings to couples in which both partners are carriers. We have argued that the church should impose these burdens on couples only if doing so promotes a sufficiently compelling goal and there is no less burdensome way to achieve this goal. Reducing the burden of SCA in Nigeria is a compelling goal. However, testing earlier in life offers a less burdensome and likely more effective means of achieving this goal. This suggests that the church might advocate for earlier screening for SCA and help to support these programs as they are developed. Finally, we have argued that this approach is also likely better able to promote the church’s own goals of minimizing burden on its parishioners and increasing the number of church weddings.

## Data Availability

Not applicable

## Abbreviations

DHHS: Department of Health & Human Services
NDHS: National Demographic Health Survey
NIH: National Institute of Health
SCA: Sickle cell anemia
SCD: Sickle cell disease
USA: United State of America
WHO: World Health Organization
